# The benefits and challenges of taxing sugar in a small island state: an interrupted time series analysis

**DOI:** 10.1186/s12966-022-01308-x

**Published:** 2022-06-15

**Authors:** Alexa Blair Segal, Jack Olney, Kelsey K. Case, Franco Sassi

**Affiliations:** 1 Centre for Health Economics & Policy Innovation, Department of Economics & Public Policy, Imperial College Business School, Imperial College London, South Kensington Campus, London, SW7 2AZ UK; 2 Department of Infectious Disease Epidemiology, Imperial College London, London, W2 1PG UK

**Keywords:** Economics, Diet, Healthy policy, Fiscal policy, Public health, Sugar-sweetened beverages, Obesity prevention, Diabetes prevention

## Abstract

**Background:**

Beverage and food taxes have become a popular ‘best buy’ public health intervention in the global battle to tackle noncommunicable diseases. Though many countries have introduced taxes, mainly targeting products containing sugar, there is great heterogeneity in tax design. For taxes levied as import tariffs, there is limited evidence of effectiveness in changing the price and sale of taxed products, while the evidence base is stronger for excise taxes levied as a fixed amount per quantity of product. This paper examines the effect of the Bermuda Discretionary Foods Tax, which was based on import tariff changes, on retail prices and sales of sugar-sweetened beverages (SSBs), and on selected fruits and vegetables that benefited from a tariff reduction.

**Methods:**

We used weekly electronic point-of-sale data from a major food retailer in Bermuda. We assessed historical weekly sales and price data using an interrupted time series design on 2,703 unique products between the dates of January 2018 through January 2020, covering 103 weeks.

**Results:**

By January 2020, the average price per ounce of SSBs increased by 26.0%, while the price of untaxed beverages (including waters and non-added sugar drinks) remained constant. The increasing price of SSBs was the sole observable structural driver of SSB market share, responsible for a decrease in the market share by nearly eight percentage points by the end of the study period. The subsidy on fruits and vegetables was ineffective in changing prices and sales, due to the relatively small 5% import tax decrease.

**Conclusions:**

The tax was largely passed through to consumers. However, several factors mitigated the impact of the tax on the prices paid for SSBs by consumers, including the specific design of the tax, price promotions and consumer responses. The experience of Bermuda provides important lessons for the planning of similar taxes in the future.

**Supplementary Information:**

The online version contains supplementary material available at 10.1186/s12966-022-01308-x.

## Background

A growing body of evidence shows taxing beverages with added sugar helps reduce excess sugar consumption [1]. Consumption of sugar-sweetened beverages (SSBs) is associated with a higher incidence of overweight and obesity, type 2 diabetes, cardiovascular risk factors, and dental caries [2]. The World Health Organization (WHO) has recommended countries implement fiscal measures, such as the taxation of SSBs, to reduce sugar consumption and aid in the prevention of obesity and non-communicable diseases [1, 2]. Countries have employed various taxes on SSBs to reduce consumption of high-sugar drinks, though fewer countries have implemented subsidies to encourage consumption of healthier products [3].

As of January 2020, more than 40 countries globally have implemented nation-wide SSB taxes, affecting over 2 billion people [4]. SSB taxes are designed to increase the price of SSBs, lowering demand and ultimately improving population health. However, the effectiveness of SSB taxes is dependent upon tax design, among other factors, as the design affects the ability of the tax to successfully increase the retail price of SSBs and reduce overall SSB consumption [4, 5]. Designing effective SSB taxes includes selecting which beverages should be taxed, the type of tax (excise or value added taxes, import tariffs) and the tax rate [6]. Though structured in various ways, most SSB taxes are specific excise taxes, meaning that the taxes are levied on the volume or specific nutrient content (i.e., $0.10 per litre of SSB or $0.05 per gram of sugar). At least some SSB excise taxes are designed to encourage beverage manufacturers to reformulate their products and reduce their sugar content [6]. Some taxes, however, are “ad valorem”, i.e., they are calculated as a proportion of the products’ price [7]. A possible downside of ad valorem taxes is that they may encourage consumers to opt for cheaper alternatives (i.e., brand-down switching to a cheaper item with the same amount of sugar) [7]. There is strong evidence from the literature on tobacco taxes that ad valorem tax structures are vulnerable to changes in both the producers’ and consumers’ behaviour, though this is difficult to predict [8, 9]. In a 2017 systematic review of empirical studies of health taxes, the authors found that specific taxes were associated with stronger health benefits than that of ad valorem taxes [9]. Further, a paper that evaluated the effect of a cut in value-added taxes (an additional type of ad valorem tax) on food in French restaurants observed that passthrough may be lower using value-added taxes compared to excise taxes [10]. Thus, understanding the effect of tariff SSB designs on consumer behaviour will be critical in aiding policymakers on the design of future policies, as the literature on the effects of ad valorem tax design on consumers and manufacturers is currently limited.

There is robust evidence that SSB taxes successfully increase the retail price of taxed SSBs, though disparities in tax design contribute to their effectiveness, both in increasing the retail price and reducing overall SSB consumption [4, 5]. A major problem associated with SSB taxes comes from consumers who substitute to other high-sugar non-taxed products, or consumers purchasing SSBs in neighbouring jurisdictions, where the tax does not apply [4]. This is why investigating the effects of SSB taxes in small island states is useful due to the fact that the consumers cannot buy products from neighbouring regions. In addition to this, studying the effects of SSB taxes in an island setting is important because some of the highest rates of obesity and diabetes in the world are being observed in small island jurisdictions [11]. This can be attributed to the rapid nutritional changes occurring due to the dependency on trade, which, in turn, has increased access to processed food [11].

Due to the increasing rates of obesity and diabetes, there has been a prolific rise in sugar taxes being adopted in these small island settings; 75% of the 21 Pacific Islands countries and territories have implemented an SSB policy from the years 2000 to 2019 [11]. The WHO and the Healthy Caribbean Coalition have worked to introduce these types of taxes to improve health of citizens. While there is concreate evidence for the effectiveness of SSB taxes implemented as excise taxes, there is more limited evidence of effectiveness of import tariffs, especially in the case of island nations.

Various island states that have implemented SSB taxes, though the efficacy of the SSB tax is inconsistent. For example, in 2015, Barbados – a high-income island – implemented a 10% ad valorem tax on SSBs. An evaluation of the SSB tax found that the SSB tax was associated with lowered SSB sales, though there was evidence that consumers substituted to lower-priced but equally as sugary alternatives and untaxed beverages [7]. Similar to this present study, untaxed beverages in Barbados included beverages with non-nutritive sweeteners (NNS) or artificially-sweetened beverages (ASBs), otherwise known as non-caloric sweeteners. The inclusion of NNS beverages is a controversial issue in the design of beverage taxes. While no adverse health effects of NNS have been consistently reported [12], there are concerns that NNS beverages may inadvertently increase consumers’ total caloric intake via multiple pathways – either by justifying consumption of other calorific foods or by increasing preferences for sweeter flavours [13, 14].

Our study focuses on Bermuda, where three out of four adults have overweight or obesity, and half of the population consumes at least one SSB per day [15]. The objective of this study is to evaluate the effect of the Bermuda Discretionary Foods Tax, an ad valorem import tariff, on sales and prices of select affected products. For beverages, the tax was implemented in two phases: in October 2018, the import tariff on SSBs (not including non-nutritive sweetened beverages) increased from 33.5% to 50% and six months later, in April 2019, increased to 75% [15, 16]. Beverages sweetened with non-nutritive sweeteners (i.e., diet sodas) were not included in the tax. At the same time of the first tax increase for SSBs, the Bermudian government issued a subsidy on select F&V, resulting in a reduction in duty from 5 to 0% [15, 16].

While taxing products high in sugar (or saturated fats, trans fats, salts, etc.) has been shown to raise prices, potentially deterring consumption, there is less evidence on the effect of food subsidies on prices [17]. Current evidence suggests subsidising fresh fruits and vegetables (F&V) by 10–30% is effective in promoting their consumption [17]. A recent modelling study by Blakely et al. 2020 modelled the effect of a 20% F&V subsidy on F&V purchases in New Zealand, finding that the F&V subsidy increased fruit purchasing by 16.2% and vegetable purchasing by 32.0% [18]. The subsidy was associated with beneficial substitution effects, including decreased saturated fat and salt purchasing [18]. Theoretically, subsidies on healthier alternatives, such as F&V, work in conjunction with taxes to encourage consumers to switch to healthier alternatives.

Thus, the objective of our study was to examine the impact of the Bermuda Discretionary Foods Tax on the price and purchases of selected SSBs and non-SSBs, as well as selected F&V. Our findings from the Bermuda tax are likely to be relevant to countries internationally, in addition to island states.

## Methods

**Fig. 1:**
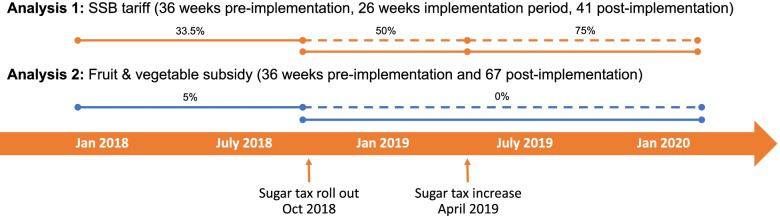
Schematic of overall analysis plan. Solid lines = observed data; dashed lines = counterfactual estimated from formerly observed data. Percentages above lines represent tax rate during each stage

The goal of this study is to examine the changes in the prices and the volume of SSBs sold and the same for F&V that were included in the subsidy from nine months before the tax was implemented, six months after the tax increased and then 10 months after the tax increased again (January 2018 until January 2020). An interrupted time series (ITS) approach was employed, allowing for the comparison of the observed changes associated with two implementation periods (only the first period for the F&V) to a counterfactual in which the intervention did not occur (Fig. 1) [19]. The full definition for the Discretionary Foods Tax is found elsewhere [15, 16] and the strengthening the reporting of observational studies in epidemiology (STROBE) checklist is in Additional file 1.

Note that the Bermuda Discretionary Foods Tax covered additional products; initially in the form of a 50% import tariff on SSBs, candies, and pure-sugar imports (raising pre-existing tariffs by varying degrees), and then in April 2019, the tax increased to 75% and the tax base was expanded to include food products containing cocoa. However, as the focus of this study was to evaluate the effect of the Tax on taxed SSBs and subsidized F&V, the other products covered by the tax were out of the scope of this project.

### Data source

Electronic point-of-sale data was provided by a major grocer in Bermuda. Depending on the vendor, this grocer had about 35%-40% of the grocery market share in Bermuda (in conversation with Z. Moniz, Manager at Lindo’s in November 2020). Data were available from January 27, 2018, to January 12, 2020, with the price and unit sales aggregated by week (1,898 size-specific beverage products and 353 size-specific produce products).

### Product categories

Products were categorised for analyses according to the definitions in the Bermuda Customs Tariff schedule (Table 1) [16]. To evaluate the impact of the subsidy on fruits and vegetables, all fruits and vegetables that were included in the subsidy were included in the analysis, as listed in Table 1. The tax may have led consumers to substitute SSBs with other beverages. Therefore, the purchasing of untaxed beverages as a possible substitute was examined (these products are referred to as “untaxed” beverages, but this does not mean the tax rate was zero; in this case, untaxed beverages included waters and non-added sugar beverages).

**Table 1:** Categories for analyses

**Overall categories**	**Tariff code**^**a**^	**Detailed Product Categories** [16]	**Explanation (Example)**	**Unique products (**^**b**^**)**
SSBs (taxed) 75%	2202.101	Waters, including mineral waters and aerated waters, containing added sugar or other sweetening matter, or flavoured “Waters, containing added sugar”	Sweetened with primarily sugar, corn syrup, fructose, etc. (Coca-Cola, Mountain Dew, Fruit punch)	1059 (331)
Non-SSBs (untaxed) 15%	2201.100	Mineral waters and aerated waters	No sugar added, still or sparkling (La Croix, Nirvana water, Perrier)	332 (115)
	2202.109	Waters, including mineral waters and aerated waters, containing added sugar or other sweetening matter, or flavoured “Other”	Sweetened with primarily NNS (Diet Coke, Diet Sprite, Diet tonic water)	357 (101)
	2202.990	Other miscellaneous drinks	Protein drinks/shakes with added sugar or NNS (PediaSure, Slimfast, Ensure)	150 (58)
Subsidy (untaxed) 5% to 0%	701.900	Potatoes, fresh or chilled: “other”		101 (31)
	704.100	Cauliflowers and headed broccoli		57 (14)
	706.101	Carrots and turnips: Turnip, yellow		7
	706.109	Carrots and turnips: “other”		5 (1)
	805.100	Citrus fruit, fresh or dried: “Oranges”		44 (6)
	808.100	Apples		139 (27)

No alcoholic beverages or dairy products were used in this analysis

^a^Tariff 2202.101 codes for SSBs, whereas tariff 2202.109 codes for ABS/NNS beverages

^b^Number of products dropped from the price analyses due to not being on market at week one or 103

Taxed beverages included beverages, aerated or non-aerated, containing added sugar or other sweetening matter (tariff code 2202.101). In this case, “other sweetening matter” is referring to other sugar substances such as high-fructose corn syrup, sucrose, fructose, etc. that are used to sweeten SSBs. Note that though some SSB taxes include 100% fruit juices even though fruit juices are not “sugar-sweetened,” the Bermuda tax did not include said fruit juices in this tax; instead, they remained at the 5% tax rate for the duration of the study.

Conversely, “untaxed” beverages in this analysis included three tariff codes: tariff 2201.100 included mineral waters and aerated waters with no added sugars or NNS; tariff 2202.109 included beverages, aerated or non-aerated, containing other sweetening matter, meaning NNS such as aspartame or sucralose; and tariff 2202.990 included “other miscellaneous drinks” which coded for protein shakes and drinks. While some of these products do include added sugar, these products are not included in the tax because they are also supplying protein and/or meal replacements, and as such, provide more nutrition than a purely SSB with little to no nutrition aspect.

### Outcome measures

The primary outcome was the price of taxed and non-taxed products, as this provided an opportunity to evaluate the price elasticity for SSBs. The secondary outcome was sales, volume in ounces sold *per capita*, of SSBs, and for F&V, volume in pounds sold *per capita*, as defined by the tariff codes. Sales are reported *per capita* to facilitate interpretation; population size was extracted from the World Bank database [20]. To understand the dynamics associated with the weekly sales of SSBs, the tertiary outcome was weekly market share of SSBs sold compared to total ounces of both taxed SSBs and untaxed beverages sold.

### Overall analysis approach

An ITS design is a quasi-experimental time series analysis using longitudinal data with a clearly defined point of intervention (Fig. 1 schematic). For beverages specifically, the two aforementioned implementation phases are considered. Untaxed beverages, including waters and other non-SSB drinks remained at the 15% duty rate. Concurrently with the rollout of the tax, duty was cut for specific F&Vs, decreasing from 5 to 0%.

### Primary analysis

#### Beverages

To examine the effect of the tax on the price of beverage products, price patterns over the study period were inspected. The effect of SSBs (tariff 2202.101) was estimated separately from the other beverage options as duty on products in this tariff code increased significantly over the course of the study period, from 33.5% to 50% in week 36, and to 75% in week 62. To account for the differing size of products, the price per ounce of each product sold was calculated, then summed across tariff codes (see Additional file 2: Appendix A). To estimate whether the increase in duty on SSBs had any effect on the price of non-SSBs, the products from the tariff codes 2201.100, 2202.109 and 2202.990 were aggregated (identified in Table 1).

To examine the effect of the tax on consumer sales, we followed the ITS model outlined by Alvarado et al*.* [7] and used an Ordinary Least Squares regression model, assuming a normally distributed outcome, separately for SSBs, and added an additional intervention period to capture the two increases in tax, while controlling for price per ounce, holidays (Christmas, Easter and the Bermuda Cup Match, a 2-day Bermudian holiday which primarily celebrates cricket), season, and temperature (Additional file 2: Appendix B). Note that we discussed the inclusion of confounders with a variety of stakeholders in Bermuda. The stakeholders stressed that population changes and economic changes would not be relevant in our study. Instead, they stressed the importance of controlling for temperature (as this coincides with tourism season, where the Bermudian population temporarily rises for a short duration). If we controlled for both tourism season and temperature, issues of collinearity arose. Finally, to understand if sales of SSBs changed over the study period, a model to investigate the proportion of taxed SSBs sold compared to all beverages was constructed (Additional file 2: Appendix C).

#### Fruits and vegetables

To examine the effect of the tax on the price of duty-free F&V products, the pattern of price over the study period was investigated. The effect was aggregated over six tariff codes (701.900, 704.100, 706.101, 706.109, 805.100 and 808.100 in Table 1) coding for produce affected by the tax, decreasing from 5 to 0% at week 36. To account for the differing size of the products, the price per ounce of the product sold was calculated, then totaled by the tariff code. To examine if the reduction in duty on specific produce affected sales, the same model outlined above for beverages was followed (Additional file 2: Appendix B and C).

## Results

### Effects on prices

#### Beverage prices

**Fig. 2:**
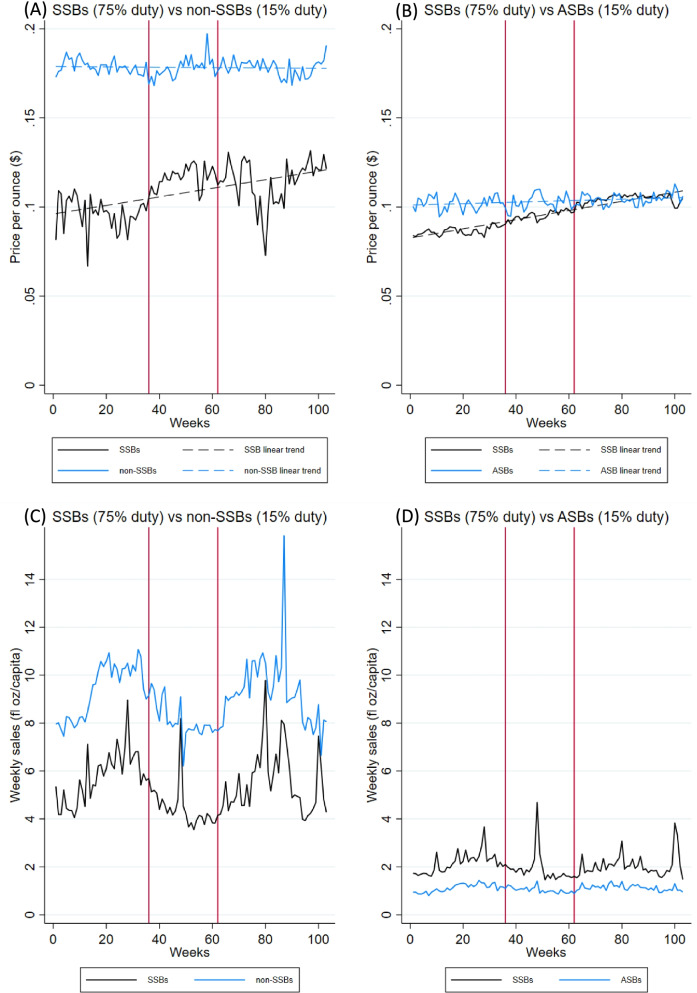
Price and sales per capita per week for beverages. Week one corresponds to the week of January 27, 2018; week 103 corresponds to January 12, 2020. First vertical red line at week 36 corresponds to October 6, 2018, the week that the first implementation of the tax begun; the vertical red line at week 62 corresponds to April 6, 2019, the second implementation period of the tax. In panels **A** and **C**, non-SSBs include waters, ASBs and other drinks; in panels **B** and **D**, ASBs were limited to soft drinks, namely diet sodas

Price variations of products consistently on the market were separated from variations due to new products entering the market (cheaper or more expensive than pre-existing ones), or those being discontinued. As a result, products not on the market at week one or week 103 were dropped, equating to 800 products of the 2,703 beverages included in this analysis, i.e., 30% of products, and 10% of sales. The average price per ounce of non-SSBs remained higher than that of SSBs throughout the study period, fluctuating between $0.16 to $0.20 per ounce (Fig. 2, Panel A). Compared to non-SSBs, the price of SSBs was more dynamic, ranging from $0.06 to $0.13 per ounce. Large decreases were observed in the average price per ounce of SSBs around week 11 (with Easter 2018 falling in week 10) and week 79 (coinciding with the 2019 Cup Match on 1st and 2nd August 2019), suggesting intense price promotions around these times, but also that consumers may purchase larger shares of lower price products when there are peaks in demand.

Overall, the average price per ounce for non-SSB products remained constant over the two-year period. Conversely, the average price per ounce of SSBs increased by 26.0% over the two years. Despite substantial duty increases, there was no evidence of a large one-time increase in price when duty rates were changed. Instead, a gradual price increase of SSBs was observed, in line with pricing practices at the retail level (in conversation with Z. Moniz, Manager at Lindo’s in November 2020). This meant the average price of taxed beverages (over one thousand products) increased gradually, following new shipments, as the retailer was able to sell the products they had at the pre-tax price first. In this way, there was a gradual pass through of the tax to consumers instead of a sudden change.

The average price of non-SSBs remained substantially higher than SSB products throughout the observation period, despite the increase in SSB prices. However, this differs when only carbonated soft drinks (i.e., sodas/fizzy drinks) are considered, accounting for half of SSB sales (Fig. 2, Panel B). The average price of sugar-sweetened sodas increased by approximately 33% over the study period, matching the price of artificially sweetened (diet) sodas by the end of the study period.

#### Fruit and vegetable prices

**Fig. 3:**
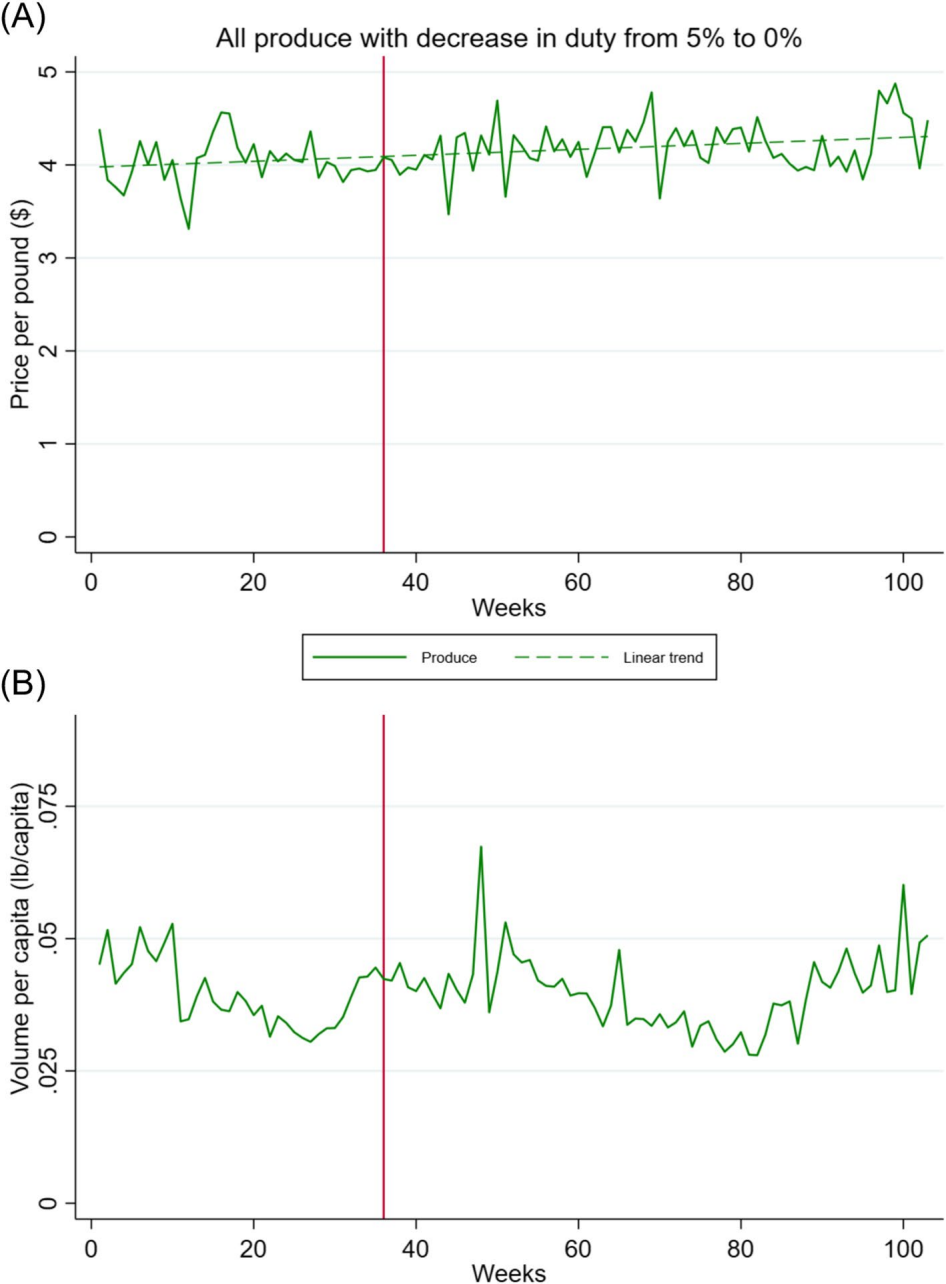
Price and sales per capita per week for fruit and vegetables. Week one corresponds to the week of January 27, 2018; week 103 corresponds to January 12, 2020. Vertical red line at week 36 corresponds to October 6, 2018, the week that implementation of the tax begun. Produce (F&V) includes potatoes, cauliflowers, broccoli, carrots, turnips, oranges and apples

Products not on the market in weeks one and 103 were dropped, thus dropping 86 (24%) of the 353 unique F&V products. After dropping these products, the average price per pound fluctuated between $3.30 to $4.90 (Fig. 3, Panel A). The average price of the subsidised F&V products increased by 2.2% over the study period. The prices of F&V are strongly driven by market conditions and are subject to significant seasonal variations. Some products included in this duty cut, such as carrots, are largely produced locally, so the duty cut would not be expected to affect the price or sales of the product. A few peaks and troughs were observed over our two-year period. For instance, the large peak around week 94 coincides with the start of orange season, when the price increases until the supply of oranges is high (in conversation with Z. Moniz, Manager at Lindo’s in November 2020).

### Effects on sales

#### Beverage sales

Trends in beverage sales show significant weekly variation, especially for SSBs, with a clear seasonal pattern and peaks corresponding to holidays and major sport events (Fig. 2, Panel C). Our initial assumption was that grocery prices would be determined exogenously, as Bermuda is a small market and manufacturers are unlikely to adapt pricing strategies to expected fluctuations in demand. However, during the analysis we observed price promotions were often timed to coincide with events that are known to be associated with a stronger than usual demand for beverages, particularly SSBs. This form of endogeneity was addressed by adjusting for major events (holidays and popular sporting events) typically associated with steep increases in demand.

Results from the main ITS model designed to assess changes in SSB sales trends following the phased introduction of the tax, accounting for price changes and adjusting for temperature, major holidays and sporting events and seasonality, produced the results summarized in Table 2. These results show that the adjustments made capture most of the weekly variations in beverage sale volumes and point to a strong link between the average price paid by consumers for SSBs and the sale volumes recorded each week. The coefficient of the price variable (-59.48, *p* < 0.001), suggests a price elasticity around one for SSBs during the period of observation, with a proportionate reduction in consumption approximately equivalent to the proportionate price increase. The model shows prior to the first implementation period, SSB sales were increasing significantly every week by 0.024 oz per person. In the first week of the intervention at week 36, there was a slight decrease in SSB sales of 0.23 oz, followed by a significant decrease in purchases relative to the pre-implementation by 0.037 oz per capita per week (*p* = 0.026). This effect was additional to the price effect and might reflect the prominence of the tax in the media and public debate around the time of its initial implementation. However, following the second stage of implementation, when import tariffs on SSBs were further increased, no significant effects beyond that of prices were observed. These results do not highlight further statistically significant trends following the phased implementation of the tax as the second post-intervention trend was negligible in size and not statistically significant.

**Table 2:** Interrupted time series results for SSBs

	**(1)**		**(2)**	
	**Volume per capita**	**Volume per capita**^**a**^	**Market share**	**Market share**^**a**^
Trend prior to Tax 1	0.024* [0.00,0.05]	-0.005 [-0.04,0.03]	0.001** [0.00,0.00]	0.000 [-0.00,0.00]
Level change after Tax 1	-0.227 [-0.81,0.36]	-0.649 + [-1.38,0.09]	0.009 [-0.01,0.03]	-0.007 [-0.04,0.02]
Trend after Tax 1, before Tax 2	-0.037* [-0.07,-0.00]	0.003 [-0.05,0.05]	-0.002** [-0.00,-0.00]	-0.001 [-0.00,0.00]
Level change after Tax 2	0.086 [-0.38,0.55]	-0.291 [-0.84,0.25]	0.005 [-0.01,0.02]	-0.010 [-0.04,0.02]
Trend after Tax 2	0.019 + [-0.00,0.04]	0.009 [-0.02,0.04]	0.002** [0.00,0.00]	0.001 [-0.00,0.00]
Price per ounce ($)	-59.476*** [-78.21,-40.74]		-2.330*** [-2.91,-1.75]	
Holidays	1.334** [0.37,2.29]	1.618*** [0.69,2.55]	0.045* [0.01,0.08]	0.056*** [0.02,0.09]
Season	0.246 [-0.07,0.56]	0.273 [-0.13,0.68]	-0.007 [-0.02,0.01]	-0.006 [-0.02,0.01]
Average Temperature (Celsius)	0.044 + [-0.00,0.09]	0.158*** [0.08,0.24]	-0.002 [-0.00,0.00]	0.003 [-0.00,0.01]
Overall trend after Tax 1	-0.012 [-0.03,0.01]	-0.002 [-0.03,0.02]	-0.001* [-0.00,-0.00]	-0.000 [-0.00,0.00]
Overall trend after Tax 2	0.007 [-0.00,0.02]	0.007 [-0.01,0.02]	0.001* [0.00,0.00]	0.001 + [-0.00,0.00]
Constant	9.436*** [7.29,11.58]	1.534* [0.02,3.05]	0.617*** [0.54,0.70]	0.308*** [0.24,0.37]

Model 1 the outcome is weekly volume per capita (in ounces)

Model 2 the outcome is weekly market share of SSBs of all beverages (Additional file 2: Appendix C)

+ *p* < 0.1, *p < 0.05, **p < 0.01, ***p < 0.001

^a^Without price in the model

A further ITS model was developed to test whether the estimated reduction in SSB sales was associated with substitution towards non-taxed beverages, displayed in Table 2. After accounting for price changes (which had a clear and statistically significant association with SSB market share), adjusting for temperature, season and major holidays and sport events, the model did not highlight further meaningful or statistically significant changes in the market share of SSBs. The increasing price of SSBs were the sole structural driver of SSB market share, responsible for a decrease in the market share by nearly eight percentage points by the end of the study period, which is only marginally larger than the decrease that would be expected based solely on the reduction in SSB sales. We conclude the phased introduction of the tax in Bermuda reduced SSB sales fully in line with expectations but did not lead to a meaningful substitution of SSBs with non-taxed beverages. This is in keeping with the consistently higher average price of non-taxed beverages throughout the observation period.

#### Fruit and vegetable sales

Trends in weekly sales of F&V whose import tariff was reduced in October 2018 are shown in Fig. 3, Panel B. Results from the main ITS model designed to assess changes in F&V sales trends following the phased introduction of the subsidy, after accounting for price changes and adjusting for temperature, major holidays and sporting events and seasonality, produced the results summarized in Additional file 2: Appendix D. Trends display clear seasonal patterns and significant short-term fluctuations, but not necessarily due to the same factors as for beverages. Given the trends observed in F&V prices during the period of observation, we did not further investigate the effects of the tariff reduction on F&V sales.

## Discussion

This study presents evidence of the sales and price effects of the Bermuda tax on beverages as well as the effect of the subsidy on produce items. Our analysis indicates the average price of SSBs increased by 26.0% over the two-year study period (January 2018 to January 2020), while the average price of non-SSB products remained constant. The tax was largely passed through to consumers, but due to its design, price promotions, and consumer responses, the prices paid by consumers for SSBs did not increase proportionally to the tax. For several weeks following the second tax increase in 2019, consumers purchased SSBs at significantly lower than average prices, likely reflecting a combination of price promotions (e.g., coinciding with large events associated with increased SSB demand) and the action of consumers ‘trading down’ to cheaper products, a known side effect of *ad-valorem* taxes.

A clear consumer response to SSB price changes over time is observed, suggesting a price elasticity in line with those found in other high-income settings as suggested by a meta-analysis (around one) [21]. Our results indicate SSB sales were down by one quarter (26.0%), compared to where they would have been had prices not increased following the introduction of the tax. After accounting for a wide range of factors, including price, temperature, season, holidays, and sporting events, we did not reveal further underlying trends and effects linked with the two tax increases.

When considering the linked tax reduction on F&V products, a different conclusion is drawn. Due to the relatively modest decrease in duty from 5 to 0%, together with the typically large fluctuations in base price (due to market factors), prices increased consistently throughout the study period, and the tax had no significant impact on the sales of F&V. This findings is in line with previous findings that if a tax rate is too low (in this case, reduction in tax is too low), the health impact is null [18]. A study based on US data suggests that a subsidy needs to be set at 10% to have a meaningful impact in terms of reducing cardiovascular disease burden and reducing socio-economic disparities [22]; while a modelling study from New Zealand suggests a subsidy of 20% on F&V is needed to encourage purchasing of F&V [18].

Compared to other island nations, where issues of cross-border shopping are not an issue, we find disparate results. Barbados employed a 10% ad valorem excise tax structure, whereby there was a uniform tax rate (not tiered), and the tax was not based on sugar content. An evaluation of the Barbados SSB tax shows that the weekly sales of SSBs decreased significantly following the tax, with weekly sales of non-SSBs increasing significantly [7]. However, the main analyses did not control for the price of the products; in a sensitivity analysis, they stratify by price tercile and find evidence of substitution to cheaper SSBs [7]. Thus, a reason why the SSB tax in Barbados may have been more effective in encouraging consumers to purchase the non-SSB products (or switching to cheaper SSBs) was because the price of the non-SSB alternatives was cheaper, comparatively, than the non-SSB alternatives in Bermuda [7].

The main limitation of our analysis is that sales data were obtained from a single source. This limitation results from the need for item-level data on sales and price histories over several years for our analysis. The relatively short duration of data, combined with several big events occurring in Bermuda (e.g., Cup Match), led to a noisy dataset requiring several covariates to isolate the effect of the tax. Also, the lack of individual-level data on changes in consumption meant that patterns needed to be inferred from aggregate sales data, allowing us to only evaluate store-level changes in sales. This also means that we were unable to assess changes in different age groups or different income levels. After consultations with the retailer and several government and beverage and food stakeholders in Bermuda, we have no reason to believe the size of the client base served by the retailer may have changed in a meaningful way and are confident tourist flows were not affecting sales in a significant way.

Another limitation to our analysis is that we have not assessed substitution of SSBs with other discretionary foods. The evidence of substitution to other food products is less clear for health-related food taxes because changes in substitutions and complementary foods are difficult to predict. However, the evidence suggests that these substitution effects are “more likely to happen with taxes on specific nutrients rather than broader food categories because the complexity of a nutrient tax makes it harder to model and evaluate how the overall diet will be affected” [23]. In the case of Bermuda, substitutions towards foods containing sugar were limited by the application of the tax to selected foods, as well as beverages. Substitutions that are of a greater concern with SSB taxes are those towards untaxed beverages containing sugar. However, because the Bermuda Discretionary Foods Tax included all SSB products (except for juices), substitutions to other SSBs may be limited. Therefore, overall, we are reasonably confident that consumers in Bermuda had limited opportunities to substitute SSBs with other products containing sugar.

A final limitation is the lack of control group in our analyses, specifically in the F&V model. We had tried to find a suitable control group, from which we hypothesised that vinegar or hair shampoos/conditioners would be suitable as a control group for our analysis. However, when we went back our data supplier, we had issues with data availability. Essentially, the store destroys all data for the price and sales of their stores after two years. By the time we started this project, cleaned the data and began conducting analyses, we learned it was too late to request the appropriate data on controls as the data for our study period would be destroyed.

## Conclusions

Based on this analysis, the tax in Bermuda increased prices and decreased purchases SSBs in line with expectations and with experience in other settings where similar taxes have been applied [21]. To date, Bermuda has implemented among the highest import tariffs for sugary confection products [4]. However, we show non-SSBs remained just as expensive as the targeted SSB products. To encourage consumers to switch to healthier products, future policies should work to lower the prices of healthier alternatives. Further, the subsidy for the F&V was shown ineffective in this study, as the 5% decrease in tax did not effectively reduce the price of selected F&V included in the subsidy. Further policy measures should increase and expand this subsidy to encourage consumers to buy F&V, for example, by providing healthy food vouchers for low-income consumers. Finally, based on our observation that price promotions on SSBs seriously affected sales of SSBs, we suggest that policy makers keep this in mind when designing future fiscal policies to ensure that price promotions are not allowed on these high-sugar products.

Our analyses show when combining the findings on trends in purchases of SSBs and on the relative market shares of taxed and non-taxed beverages, very limited substitution took place between the SSB and non-SSB categories, and the decreased market share of SSBs over the period of observation largely reflects their decreased consumption following the introduction of the tax. Our study suggests the Bermuda Discretionary Foods Tax has been successful in raising the prices of targeted items, but more opportunity for success is possible if healthy substitutes to SSBs were lowered in price.

## Supplementary Information

**Additional file 1.** STROBE Checklist.

**Additional file 2.** Appendices.

## Data Availability

The datasets generated and analysed during the current study are not publicly available due to privacy reasons, because the data belongs to Zach Moniz (zach@lindos.bm), Manager at Lindo’s, a competitive retailer in Bermuda.

## Abbreviations

SSBs: Sugar-sweetened beverages
WHO: World Health Organisation
NNS: Non-nutritive sweeteners
ASB: Artificially-sweetened beverages
F&V: Fruits and vegetables
ITS: Interrupted time series
STROBE: Strengthening the reporting of observational studies in epidemiology
