# Supplementary material for: The benefits and challenges of taxing sugar in a small island state: an interrupted time series analysis
Source: Int J Behav Nutr Phys Act. 2022 Jun 15;19:69. doi: 10.1186/s12966-022-01308-x (PMC9202202; doi:10.1186/s12966-022-01308-x)
Supplement: Supplementary file 1 — Additional file 1. STROBE Checklist. [file 12966_2022_1308_MOESM1_ESM.docx]

Additional file 1: STROBE statement

STROBE Statement—checklist of items that should be included in reports of observational studies.

|  | Item No | Recommendation | Section in text |  |
| --- | --- | --- | --- | --- |
| Title and abstract | 1 | (*a*) Indicate the study’s design with a commonly used term in the title or the abstract | Lines 1-2 |  |
|  |  | (*b*) Provide in the abstract an informative and balanced summary of what was done and what was found | Lines 21-42 |  |
| Introduction | | |  |  |
| Background/rationale | 2 | Explain the scientific background and rationale for the investigation being reported | Lines 48-125 |  |
| Objectives | 3 | State specific objectives, including any prespecified hypotheses | Lines 126-127 |  |
| Methods | | |  |  |
| Study design | 4 | Present key elements of study design early in the paper | Lines 131-136 |  |
| Setting | 5 | Describe the setting, locations, and relevant dates, including periods of recruitment, exposure, follow-up, and data collection | Lines 131-136, 139-144, 16-150; Figure 1 |  |
| Participants | 6 | (*a*) *Cohort study*—Give the eligibility criteria, and the sources and methods of selection of participants. Describe methods of follow-up  *Case-control study*—Give the eligibility criteria, and the sources and methods of case ascertainment and control selection. Give the rationale for the choice of cases and controls  *Cross-sectional study*—Give the eligibility criteria, and the sources and methods of selection of participants | Lines 131-136, 139-144, 16-150; Table 1 (between lines 173 and 174) |  |
|  |  | (*b*) *Cohort study*—For matched studies, give matching criteria and number of exposed and unexposed  *Case-control study*—For matched studies, give matching criteria and the number of controls per case | N/A. |  |
| Variables | 7 | Clearly define all outcomes, exposures, predictors, potential confounders, and effect modifiers. Give diagnostic criteria, if applicable | Lines 152-182, Table 1 (between lines 173 and 174); Additional file 2, Appendix A-C (lines 8-48) |  |
| Data sources/ measurement | 8* | For each variable of interest, give sources of data and details of methods of assessment (measurement). Describe comparability of assessment methods if there is more than one group | Lines 152-181; Additional file 2, Appendix A-C (lines 8-48) |  |
| Bias | 9 | Describe any efforts to address potential sources of bias | Lines 184-222; Additional file 2, Appendix A-C (lines 8-48) |  |
| Study size | 10 | Explain how the study size was arrived at | Lines 146-150; Table 1 (between lines 173 and 174) |  |
| Quantitative variables | 11 | Explain how quantitative variables were handled in the analyses. If applicable, describe which groupings were chosen and why | Lines 152-181; Table 1 (between lines 173 and 174), Additional file 2, Appendix A-C (lines 8-48) |  |
| Statistical methods | 12 | (*a*) Describe all statistical methods, including those used to control for confounding | Lines 184-222; Additional file 2, Appendix A-C (lines 8-48) |  |
|  |  | (*b*) Describe any methods used to examine subgroups and interactions | Additional file 2, Appendix A-C (lines 8-48) |  |
|  |  | (*c*) Explain how missing data were addressed | Lines 226-230, 259-261 |  |
|  |  | (*d*) *Cohort study*—If applicable, explain how loss to follow-up was addressed  *Case-control study*—If applicable, explain how matching of cases and controls was addressed  *Cross-sectional study*—If applicable, describe analytical methods taking account of sampling strategy | Lines 152-181; Additional file 2, Appendix A-C (lines 8-48) |  |
|  |  | (*e*) Describe any sensitivity analyses | Additional file 2, Appendix C (lines 36-48) |  |
| Results | | |  |  |
| Participants | 13* | (a) Report numbers of individuals at each stage of study—eg numbers potentially eligible, examined for eligibility, confirmed eligible, included in the study, completing follow-up, and analysed | Table 1 (between lines 173 and 174); Lines 226-230, 259-261 |  |
|  |  | (b) Give reasons for non-participation at each stage | Lines 146-150, 226-230, 259-261 |  |
|  |  | (c) Consider use of a flow diagram | N/A. |  |
| Descriptive data | 14* | (a) Give characteristics of study participants (eg demographic, clinical, social) and information on exposures and potential confounders | Lines 226-230, 259-261 |  |
|  |  | (b) Indicate number of participants with missing data for each variable of interest | Lines 226-230, 259-261 |  |
|  |  | (c) *Cohort study*—Summarise follow-up time (eg, average and total amount) | N/A. |  |
| Outcome data | 15* | *Cohort study*—Report numbers of outcome events or summary measures over time | N/A. |  |
|  |  | *Case-control study—*Report numbers in each exposure category, or summary measures of exposure | N/A. |  |
|  |  | *Cross-sectional study—*Report numbers of outcome events or summary measures | Lines 226-230, 259-261 |  |
| Main results | 16 | (*a*) Give unadjusted estimates and, if applicable, confounder-adjusted estimates and their precision (eg, 95% confidence interval). Make clear which confounders were adjusted for and why they were included | Lines 226-327; Figure 2, Figure 3, Table 2 (between lines 313 and 317); Additional file 2, Appendix B-D (lines 14-55) |  |
|  |  | (*b*) Report category boundaries when continuous variables were categorized | Table 1 (between lines 173 and 174) |  |
|  |  | (*c*) If relevant, consider translating estimates of relative risk into absolute risk for a meaningful time period | N/A. |  |
| Other analyses | 17 | Report other analyses done—eg analyses of subgroups and interactions, and sensitivity analyses | Lines 301-312; Additional file 2, Appendix C (lines 36-48) |  |
| Discussion | | |  |  |
| Key results | 18 | Summarise key results with reference to study objectives | Lines 329-344, 395-399 |  |
| Limitations | 19 | Discuss limitations of the study, taking into account sources of potential bias or imprecision. Discuss both direction and magnitude of any potential bias | Lines 364-393 |  |
| Interpretation | 20 | Give a cautious overall interpretation of results considering objectives, limitations, multiplicity of analyses, results from similar studies, and other relevant evidence | Lines 329-399 |  |
| Generalisability | 21 | Discuss the generalisability (external validity) of the study results | Lines 395-413 |  |
| Other information | | |  |  |
| Funding | 22 | Give the source of funding and the role of the funders for the present study and, if applicable, for the original study on which the present article is based | Lines 434-438 |  |

*Give information separately for cases and controls in case-control studies and, if applicable, for exposed and unexposed groups in cohort and cross-sectional studies.

Note: An Explanation and Elaboration article discusses each checklist item and gives methodological background and published examples of transparent reporting. The STROBE checklist is best used in conjunction with this article (freely available on the Web sites of PLoS Medicine at http://www.plosmedicine.org/, Annals of Internal Medicine at http://www.annals.org/, and Epidemiology at http://www.epidem.com/). Information on the STROBE Initiative is available at [www.strobe-statement.org](http://www.strobe-statement.org).
