# Supplementary material for: The benefits and challenges of taxing sugar in a small island state: an interrupted time series analysis
Source: Int J Behav Nutr Phys Act. 2022 Jun 15;19:69. doi: 10.1186/s12966-022-01308-x (PMC9202202; doi:10.1186/s12966-022-01308-x)
Supplement: Supplementary file 2 — Additional file 2. Appendices. [file 12966_2022_1308_MOESM2_ESM.docx]

Additional file 2: Appendices

Contents

[Appendix A 1](#_Toc95319650)

[Appendix B 1](#_Toc95319651)

[Appendix C 2](#_Toc95319652)

[Appendix D 2](#_Toc95319653)

## Appendix A

To calculate the price per ounce for the beverage analysis, we calculated the following:

$${Price per ounce}_{w}=\frac{Total sales \left( in \$ \right) per product/week}{Total ounces sold per product/week}$$

To calculate the price per pound for the fruit and vegetable analysis, we calculated the following:

$${Price per pound}_{w}=\frac{Total sales \left( in \$ \right) per product/week}{Total pounds sold per product/week}$$

## Appendix B

Our model specification for beverages is as follows:

$${oz/capita}_{w}= \beta_{0}+ \beta_{1}T_{w}+\beta_{2}X_{w}+ {\beta_{3}T_{w}X_{w}+\beta_{4}{T2}_{w}+\beta_{5}{X2}_{w}+\beta_{6}{T2}_{w}{X2}_{w}+\beta}_{7}{Holidays}_{w}+\beta_{8}{Temp}_{w}+\beta_{9}{Season}_{w}+\beta_{10}{Price}_{w}+\varepsilon_{w}$$

where oz/capita is the outcome of interest, ounces sold per week per capita in Bermuda (Bermudian population was 62,756 in 2018), aggregated at equally spaced time periods, *w,* week, $T_{w}$is the time since the start of our study period (week 1), $X_{w}$ denotes an indicator for the period after the first tax implementation (preintervention 0, otherwise 1). The variable ${T2}_{w}$is the time since the start of our study period (week 1) to week 62, $X_{w}$ denotes an indicator for the period after the second tax implementation at week 63 (preintervention 0, otherwise 1). ${Holidays}_{w}$denotes the vector of indicators for Easter, Christmas and Cup Match, ${Temp}_{w}$denotes the average weekly temperature and $\varepsilon$ represents the error term. Cup Match is an annual cricket event celebrated in Bermuda over two national holidays in summer, with fun events planned all weekend. We also controlled for the ${Season}_{w}$to control for any spill over effects of tourism (though we are told these effects would be minimal via personal communication with retailers) and ${Price}_{w}$, the average price per ounce of the product.

$${lb/capita}_{w}= \beta_{0}+ \beta_{1}T_{w}+\beta_{2}X_{w}+ {\beta_{3}T_{w}X_{w}+\beta}_{7}{Holidays}_{w}+\beta_{8}{Temp}_{w}+\beta_{9}{Season}_{w}+\beta_{10}{Price}_{w}+ \varepsilon_{w}$$

where lb/capita is the outcome of interest, ounces sold per week per capita in Bermuda (Bermudian population was 62,756 in 2018), aggregated at equally spaced time periods, *w,* week, $T_{w}$is the time since the start of our study period (week 1), $X_{w}$ denotes an indicator for the period after the first tax implementation (preintervention 0, otherwise 1). The variable ${T2}_{w}$is the time since the start of our study period (week 1) to week 61, $X_{w}$ denotes an indicator for the period after the second tax implementation at week 62 (preintervention 0, otherwise 1). Again, all other covariates remained the same as the model for beverages above.

## Appendix C

To understand the dynamics associated with the weekly market share of SSBs, we first calculate the proportion of SSBs ounces sold (tariff 2202·101) compared to total ounces (tariffs 2202·101, 2201·100, 2202·109 and 2202·990):

$${Market share}_{w}=\frac{Total ounces of SSBs sold/week}{Total ounces of SSBs+nonSSBs sold/week}$$

$${Market share}_{w}= \beta_{0}+ \beta_{1}T_{w}+\beta_{2}X_{w}+ {\beta_{3}T_{w}X_{w}+\beta_{4}{T2}_{w}+\beta_{5}{X2}_{w}+\beta_{6}{T2}_{w}{X2}_{w}+\beta}_{7}{Holidays}_{w}+\beta_{8}{Temp}_{w}+\beta_{9}{Season}_{w}+\beta_{10}{Price}_{w}+ \varepsilon_{w}$$

where ${Market share}_{w}$is the outcome of interest, aggregated at equally spaced time periods, *w,* week, $T_{w}$is the time since the start of our study period (week 1), $X_{w}$ denotes an indicator for the period after the first tax implementation (preintervention 0, otherwise 1). The variable ${T2}_{w}$is the time since the start of our study period (week 1) to week 62, $X_{w}$ denotes an indicator for the period after the second tax implementation at week 63 (preintervention 0, otherwise 1). ${Holidays}_{w}$denotes the vector of indicators for Easter, Christmas and Cup Match, *Temp* denotes the average weekly temperature and $\varepsilon$ represents the error term. Cup Match is an annual cricket event celebrated in Bermuda over two national holidays in summer, with fun events planned all weekend.

## Appendix D

**Table D**: Interrupted time series results for produce.

|  | **(1)** | **(2)** |  |  |
| --- | --- | --- | --- | --- |
|  | **Volume per capita** | **Volume per capita†** |  |  |
| Trend prior to Tax 1 | -0.000 | -0.000 |  |  |
|  | [-0.00,0.00] | [-0.00,0.00] |  |  |
| Level change after Tax 1 | -0.003 | -0.002 |  |  |
|  | [-0.01,0.00] | [-0.01,0.00] |  |  |
| Price per pound ($) | -0.006*** |  |  |  |
|  | [-0.01,-0.00] |  |  |  |
| Holidays | 0.011** | 0.010* |  |  |
|  | [0.00,0.02] | [0.00,0.02] |  |  |
| Season | 0.002+ | 0.001 |  |  |
|  | [-0.00,0.01] | [-0.00,0.00] |  |  |
| Average Temperature (Celsius) | -0.001***  [-0.00,-0.00] | -0.001**  [-0.00,-0.00] |  |  |
| Overall trend after Tax 1 | 0.000 | 0.000 |  |  |
|  | [-0.00,0.00] | [-0.00,0.00] |  |  |
| Constant | 0.076*** | 0.051*** |  |  |
|  | [0.06,0.09] | [0.04,0.06] |  |  |

Model 1 the outcome is weekly volume per capita (in pounds).

† Without price in the model.

+ p<0.1, * p<0.05, ** p<0.01, *** p<0.001.
